# Resveratrol-Induced Xenophagy Promotes Intracellular Bacteria Clearance in Intestinal Epithelial Cells and Macrophages

**DOI:** 10.3389/fimmu.2018.03149

**Published:** 2019-01-14

**Authors:** Jana Al Azzaz, Aurélie Rieu, Virginie Aires, Dominique Delmas, Johanna Chluba, Pascale Winckler, Marie-Agnès Bringer, Jérémy Lamarche, Dominique Vervandier-Fasseur, Frédéric Dalle, Pierre Lapaquette, Jean Guzzo

**Affiliations:** ^1^AgroSup Dijon, PAM UMR A 02.102, University Bourgogne Franche-Comté, Dijon, France; ^2^University of Bourgogne-Franche Comté, Dijon, France; ^3^INSERM U1231, Lipids, Nutrition Cancer, Dijon, France; ^4^Research Team CADIR, Cancer and Adaptative Immune Response, Dijon, France; ^5^UFR SVTE—UFR Sciences de la Vie, de la Terre et de l'Environnement, Université de Bourgogne Franche-Comté, Dijon, France; ^6^Dimacell Imaging Facility, AgroSup Dijon, University Bourgogne Franche-Comté, Dijon, France; ^7^AgroSup Dijon, CNRS, INRA, Centre des Sciences du Goût et de l'Alimentation, Université Bourgogne Franche-Comté, Dijon, France; ^8^Institut de Chimie Moléculaire de l'Université de Bourgogne (ICMUB-UMR CNRS 6302), Université of Bourgogne, Dijon, France

**Keywords:** autophagy, resveratrol, xenophagy, salmonella, AIEC

## Abstract

Autophagy is a lysosomal degradation process that contributes to host immunity by eliminating invasive pathogens and the modulating inflammatory response. Several infectious and immune disorders are associated with autophagy defects, suggesting that stimulation of autophagy in these diseases should be beneficial. Here, we show that resveratrol is able to boost xenophagy, a selective form of autophagy that target invasive bacteria. We demonstrated that resveratrol promotes *in vitro* autophagy-dependent clearance of intracellular bacteria in intestinal epithelial cells and macrophages. These results were validated *in vivo* using infection in a transgenic GFP-LC3 zebrafish model. We also compared the ability of resveratrol derivatives, designed to improve the bioavailability of the parent molecule, to stimulate autophagy and to induce intracellular bacteria clearance. Together, our data demonstrate the ability of resveratrol to stimulate xenophagy, and thereby enhance the clearance of two invasive bacteria involved life-threatening diseases, *Salmonella Typhimurium* and Crohn's disease-associated Adherent-Invasive *Escherichia coli*. These findings encourage the further development of pro-autophagic nutrients to strengthen intestinal homeostasis in basal and infectious states.

## Introduction

Autophagy is a “self-eating” process that is essential for cellular homeostasis maintenance and involves the non-selective or selective degradation and recycling of cytoplasmic components by using the lysosomal pathway (1). Besides its well-known role in maintaining cellular energy balance, autophagy takes part in the innate and adaptive immunity responses by participating to intracellular pathogens clearance, by modulating the inflammatory responses and by promoting the lymphocytes development and functions (2, 3).

In the gastrointestinal tract, tissue homeostasis depends on complex interactions between the intestinal epithelium, the immune system and the microbiota (4). Diverse host regulatory mechanisms cooperate to maintain homeostasis in this tissue and their breakdown can drive to a chronic inflammatory state, as retrieved in inflammatory bowel diseases (IBD) (2, 5). Human genetic studies and experimental studies have largely established the importance of autophagy in maintaining gut integrity (6). Autophagy is a major host defense mechanism that handles and degrades invasive microorganisms. This selective form of autophagy is termed xenophagy (7). Mice that lack expression of the autophagy protein Atg5 specifically in intestinal epithelial cells are less effective in limiting the dissemination of invasive bacteria compared to their control littermate (8). In addition, mice with impaired autophagy are highly sensitive to dextran sulfate sodium (DSS) treatment, a chemical agent that causes colitis-like pathologies, demonstrating the role of autophagy in dampening the inflammatory responses. This is partly due to the regulation of inflammasome activation by autophagy regulation (2, 9, 10). In human, genetic risk variants for Crohn's disease (CD), one of the two major forms of IBD, have been identified in various autophagy-related genes (*Nod2, Atg16L1, Irgm, Ndp52, Ulk1, Atg4a*, and *Atg4d*) (11–13). In most cases, these risk variants encode a less functional protein (e.g., ATG16L1 and NOD2) or lead to an abnormally expressed protein (IRGM), resulting in defective clearance of invasive bacteria and exacerbated inflammatory responses (14–18). Knock-in mice expressing the CD-associated risk variant ATG16L1 T300A, which triggers only subtle changes in autophagy activity, exhibit an impaired xenophagy and morphological defects in Paneth and goblet cells, highlighting again the central role of autophagy in intestinal homeostasis (19). In light of the importance of autophagy in IBD etiology and development, nutritional and pharmacological approaches to stimulate autophagy are promising therapeutic options to explore (20). Interestingly, some drugs widely used in the treatment of CD, such as thiopurines (azathioprine and 6- mercaptopurine) and sirolimus, have already been shown to trigger autophagy, suggesting that manipulating autophagy is a part of their mode of action (21).

The benefits of a chronically-stimulated autophagy, notably on lifespan extension, have been largely reported in various model organisms (including nematodes, fruit flies, and mice) (22–25). In human, various pharmacological compounds (e.g., rapamycin, carbamazepine, and lithium chloride) have been described to stimulate autophagy (24, 26). However, they are poorly specific and present deleterious effects (e.g., autophagy-independent immunosuppressive activity of rapamycin). Pending the development of highly specific autophagy inducers, dietary approaches, such as caloric restriction, low-protein diet or diet enriched with micronutrients described to induce autophagy, might be valuable as inducers of basal autophagy with no deleterious side-effects. As an example, a clinical trial using low-protein diet to stimulate autophagy has shown promising results in the context of myopathies (27). Among micronutrients, resveratrol is a potent autophagy inducer (28). Resveratrol is a phenolic compound of the stilbene family that is present in wines and various parts of the grape, and classified as a novel food ingredient. It acts on autophagy by activating the sirtuin deacetylases and directly inhibiting the kinase mTOR, a master negative regulator of autophagy (28–30). Resveratrol-induced autophagy extends lifespan and ameliorates symptoms in various animal models of human diseases, including cardiovascular disorders, non-alcoholic fatty liver disease, spinal cord injury, and neurodegenerative diseases (31–35). Although its role as a global autophagy inducer is well-recognized, its role in the regulation of selective forms of autophagy, particularly xenophagy, has not been investigated so far.

Here, we analyzed the effect of *trans*-resveratrol (the most active form of resveratrol) molecules, on global autophagy and xenophagy *in vitro* in human epithelial cells and macrophages, and *in vivo* in a zebrafish model. We showed for the first time that *trans*-resveratrol treatment increases xenophagy against two intestinal and invasive pathogens, *S. enterica* serovar Typhimurium and Crohn's disease-associated Adherent-Invasive *Escherichia coli*, and that this is associated with a decrease in the inflammatory response of infected cells.

## Results

### *Trans*-Resveratrol Promotes Autophagy-Dependent Clearance of Intracellular Bacteria in Host Cells

**Figure 1:**
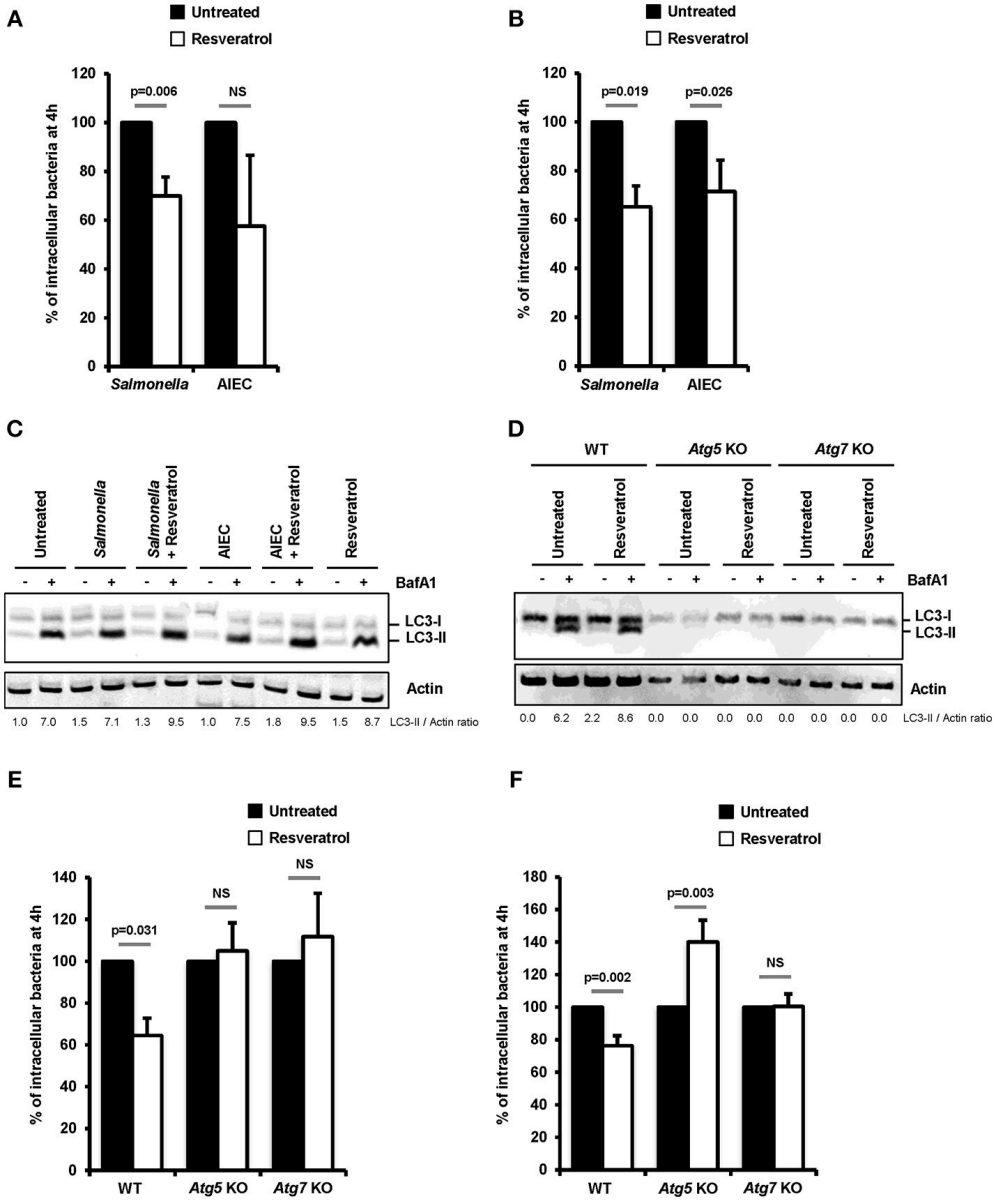
*Trans*-resveratrol promotes autophagy-dependent clearance of intracellular bacteria in host cells. **(A)** HeLa and **(B)** HCT116 epithelial cells were untreated (black bars) or pre-treated for 20 h with 10 μM resveratrol (white bars) and then infected with *Salmonella enterica* Typhimurium C5 or AIEC LF82. The number of intracellular bacteria was determined using the gentamicin protection assay at 30 min (number of bacteria internalized in the cells) and 4 h (number of intracellular bacteria that persist in cells) post-infection. Results are expressed as the number of intracellular bacteria at 4 h post infection relative to that obtained at 30 min post infection, taken as 100%. Results obtained in untreated cells were defined as 100%. Data are means ± SEM of at least three independent experiments. **(C)** HCT116 cells and **(D)** Wild-type (WT), *Atg5* knocked-out (KO) or *Atg7* KO mouse embryonic fibroblasts (MEFs), were infected with *Salmonella* or AIEC. Cells were treated with bafilomycin A1 (BafA1) at 100 nM 30 min prior infection and BafA1 was maintained in the cell medium until protein extraction, at 1 h post-infection. Quantification of LC3-II relative to Actin was done and LC3-II/Actin ratios were normalized to that obtained for untreated cells without BafA1, defined as 1.0. **(E,F)** WT, *Atg5* KO, and *Atg7* KO MEFs were infected with **(E)** *Salmonella* and **(F)** AIEC LF82. When indicated, cells were pre-treated with resveratrol at 10 μM for 20 h. The number of intracellular bacteria was determined by CFU quantification at 30 min and 4 h post-infection. Results are expressed as the number of intracellular bacteria at 4 h post infection relative to that obtained at 30 min post infection, taken as 100%. Results obtained in untreated cells were defined as 100%. Data are means ± SEM of at least three independent experiments.

Impact of *trans*-resveratrol on the ability of two intestinal pathogens, *S. enterica* serovar Typhimurium (*S. Typhimurium*) and Crohn's disease-associated Adherent-Invasive *Escherichia coli* (AIEC), to invade and persist within epithelial cells was investigated in human epithelial HeLa and HCT116 cells. Pretreatment of cells with *trans*-resveratrol at 10 μM during 20 h resulted in significant 30% (*p* = 0.006) and 35% (*p* = 0.019) decreases in the number of intracellular *S. Typhimurium* in HeLa and HCT116 cells, respectively, compared to untreated cells (Figures 1A,B). The number of intracellular AIEC bacteria was also reduced in HeLa cells (Figure 1A) and HCT116 cells (Figure 1B, *p* = 0.026) as a consequence of *trans*-resveratrol pre-treatment.

Resveratrol is known to act on many cell signaling pathways that could affect intracellular bacteria trafficking (29). Among them, resveratrol is a potent inducer of autophagy, a process already described to restrain intracellular proliferation of *Salmonella* and AIEC (8, 17, 18, 36, 37). In accordance with the literature, a 20 h *trans*-resveratrol treatment of HCT116 cells results in autophagy activation as evidenced by the higher accumulation of the LC3-II protein observed in bafilomycin A1 treated cells (LC3-II/Actin ratio = 7.0) compared to control cells (LC3-II/Actin ratio = 8.7) (Figure 1C). Of note, bafilomycin A1 is used to block the autophagy flux. This enables to visualize LC3-II protein accumulation that positively correlates with the level of autophagy activity in cells. RT-qPCR analyses show that *trans*-resveratrol treatment stimulates the transcription of a set of autophagy-related genes, including genes encoding ULK1, WIPI1, NDP52, and Optineurin, in HeLa and HCT116 cells, (Figure S1). Interestingly, pre-treatment of cells with *trans*-resveratrol also increases the autophagy response that is induced by invasive bacteria in intestinal epithelial cells as demonstrated by LC3-II accumulation (Figure 1C).

To better characterize the role of resveratrol-dependent autophagy in the clearance of intracellular bacteria, experiments were conducted in mouse embryonic fibroblasts (MEFs) knock-out for the autophagy genes *Atg5* (*Atg5* KO) or *Atg7* (Atg7 KO). As evidenced by the absence of LC3-I/II conversion both in untreated or *trans*-resveratrol-induced conditions, *Atg5* KO and *Atg7* KO cells harbor defective autophagy (Figure 1D). While *trans*-resveratrol pre-treatment of cells significantly (*p* = 0.031) decreased the load of intracellular *Salmonella* in wild-type MEFs, it has no effect in *Atg5* KO and *Atg7* KO cells (Figure 1E). Similar results were observed in *Atg7* KO cells infected with AIEC (Figure 1F). However, resveratrol treatment significantly favors AIEC bacteria intracellular persistence in *Atg5* KO MEFs, suggesting an effect related to autophagy-independent roles of Atg5 (Figure 1F). Altogether, our results demonstrated that *trans*-resveratrol induces an autophagy-dependent clearance of invasive bacteria.

### *Trans*-Resveratrol Stimulates Xenophagy

**Figure 2:**
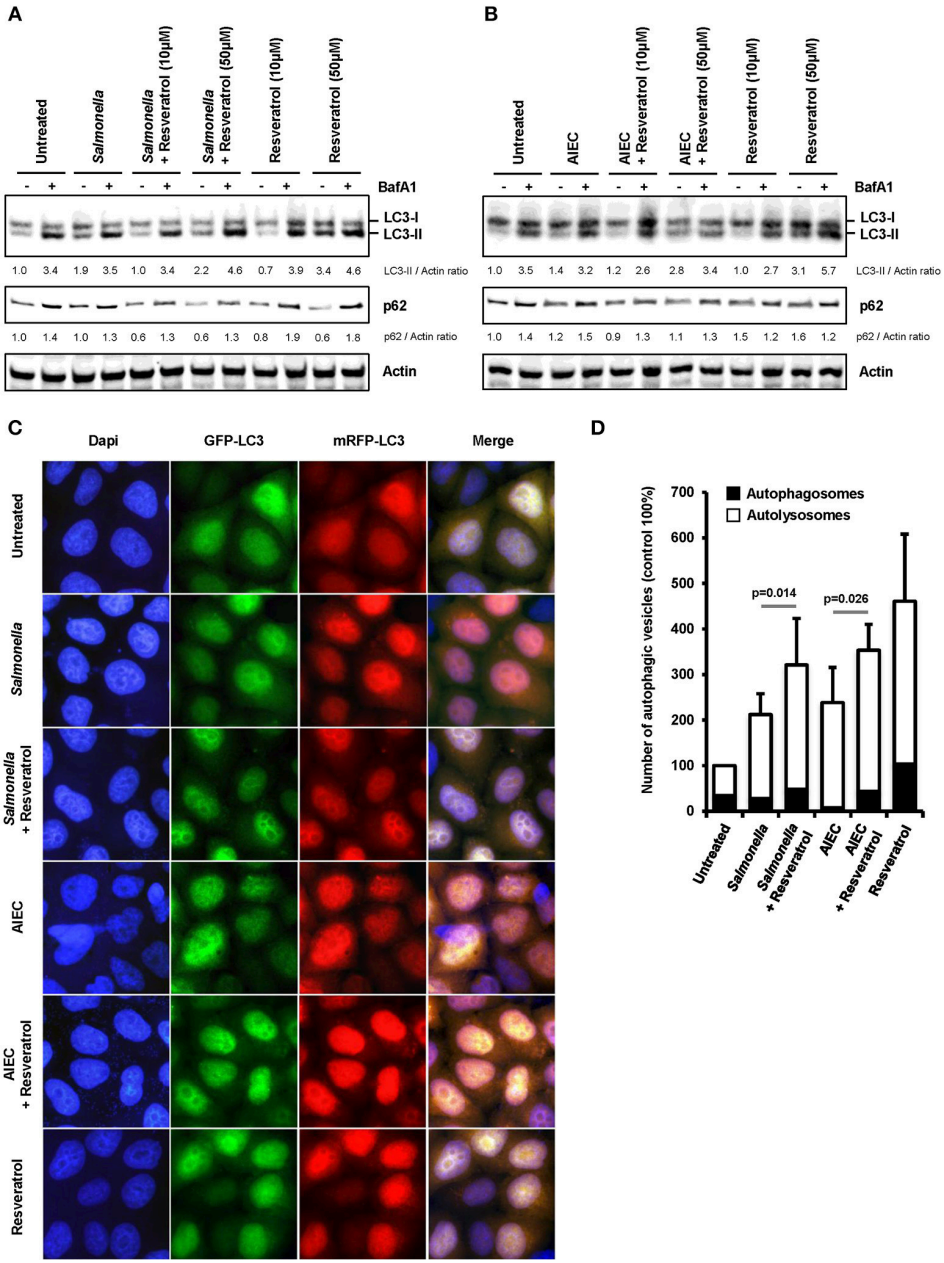
*Trans*-resveratrol exacerbates autophagy response during infection. **(A,B)** mRFP-GFP-LC3-HeLa cells were pre-treated with resveratrol at 10 or 50 μM for 20 h and then infected with **(A)** *Salmonella* and **(B)** AIEC LF82. Cells were treated with bafilomycin A1 (BafA1) at 100 nM 30 min prior infection and BafA1 was maintained in the cell medium until protein extraction, at 1 h post-infection. Quantification of p62, LC3-II, and Actin was done and the ratios of p62/ Actin and LC3-II/Actin were normalized to that obtained for untreated cells without BafA1, defined as 1.0. **(C)** Representative images of *Salmonella*- and AIEC LF82-infected mRFP-GFP-LC3-HeLa cells at 1 h post-infection, pre-treated or not for 20 h with resveratrol at 50 μM. Nuclei were labeled using DAPI (blue) **(D)** Quantification of the number of autophagosomes (RFP^+^ GFP^+^ dots) and autolysosomes (RFP^+^GFP^−^ dots) per cell using the Icy software. Results are expressed as the percentage of total autophagy vacuoles (RFP^+^ dots) per cell at 1 h post-infection, relative to that obtained in untreated cells, taken as 100%. The relative proportion of autophagosomes (RFP^+^ GFP^+^ dots, black bars) and autolysosomes (RFP^+^GFP^−^ dots, white bars) are indicated. Each value is the mean of at least three independent experiments ± SEM.

**Figure 3:**
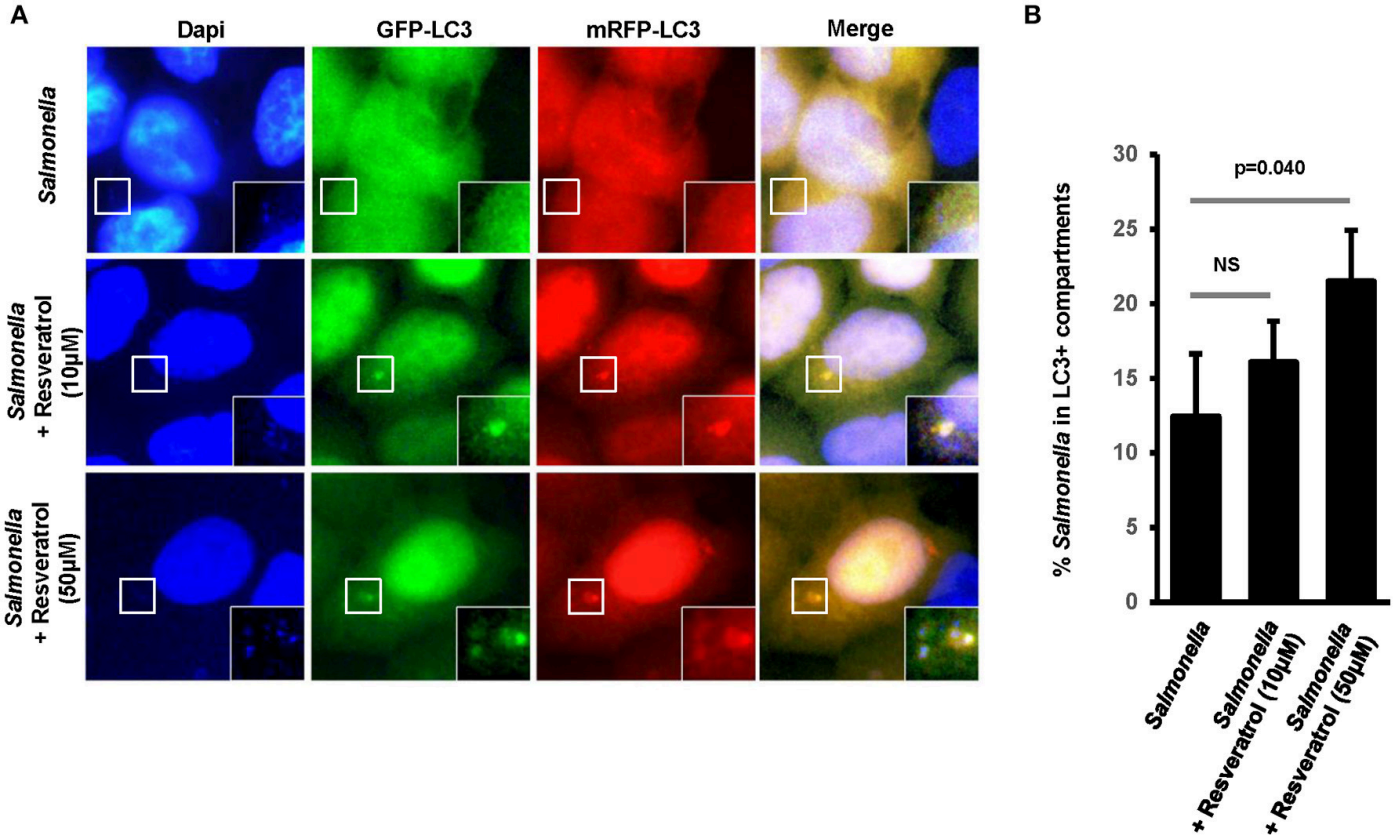
*Trans*-resveratrol increases the proportion of intracellular bacteria targeted by autophagy. **(A)** Representative images of *Salmonella*-infected mRFP-GFP-LC3-HeLa cells at 1 h post-infection, pre-treated or not with resveratrol at 10 or 50 μM for 20 h. Nuclei were labeled using DAPI (blue). White square indicates inset area. **(B)** Quantification of the percentage of *Salmonella*-enclosed in mRFP-LC3 positive vacuoles. Data are mean +/− SEM of three independent experiments. At least 50 cells were analyzed for each experiment.

We hypothesized that *trans*-resveratrol may target bacteria to autophagy. To assess this possibility, we analyzed autophagosomes in HeLa cells stably expressing a mRFP-GFP-LC3 construct that allow to discriminate early autophagic vacuoles (GFP and mRFP positive vacuoles) from acidified autolysosomes (only mRFP positive vacuoles since GFP is sensitive to acidic quenching). Cells were pre-treated or not with *trans*-resveratrol for 20 h and then infected with *Salmonella* or AIEC (Figure 2). As observed in HCT116 cells (Figure 1C), pre-treatment of HeLa cells with resveratrol leads to an increase accumulation of the LC3-II protein in *Salmonella*- and AIEC-infected cells compared to the corresponding control infected-cells (Figures 2A,B). In addition, a dose-dependent decrease in the level of p62, a LC3-binding protein degraded by autophagy, was observed in *trans*-resveratrol-treated HeLa cells, and p62 accumulates in these cells when treated by bafilomycin A1 (Figure 2A). These results confirm that autophagy is activated in epithelial cells. In agreement with the results obtained by western blot, we observed by immunofluorescence a significant increase in the total number of autophagic vacuoles (mRFP-LC3 dots) in *Salmonella* and AIEC-infected cells pre-treated by resveratrol compared to their respective control untreated cells (Figures 2C,D). Resveratrol pre-treatment favors autophagosome maturation as indicated by the increase proportion of autolysosome vesicles (only mRFP positives) in resveratrol treated-cells (Figure 2D). In *Salmonella*- and AIEC-infected cells the proportion of autophagosomes/autolysosomes is not noticeable affected by the resveratrol treatment since infection already favors autophagosome maturation compared to control cells (Figure 2D). Next, to assess whether this amplified autophagic response during infection upon resveratrol treatment favors targeting of intracellular bacteria to autophagosome, we determined the levels of *Salmonella* colocalization with LC3 in HeLa cells at 1 h post-infection (Figure 3A). Consistent with the literature (36), a subset (12%) of intracellular *Salmonella* are enclosed within LC3 positive vacuoles (Figures 3A,B). The percentage of LC3- positive vacuole containing *Salmonella* increases with the concentration of resveratrol and reach 20% when cells are treated with 50 μM resveratrol (Figure 3B). Interestingly, resveratrol treatment increases the transcription of the gene encoding p62 and NDP52 encoding two adaptor protein required to target invasive bacteria to autophagy (Figure S1) (38). Together, these results demonstrate that resveratrol increases the proportion of intracellular bacteria targeted by autophagy for degradation.

### Comparative Effects of Resveratrol Derivatives on Basal Autophagy and Xenophagy

**Figure 4:**
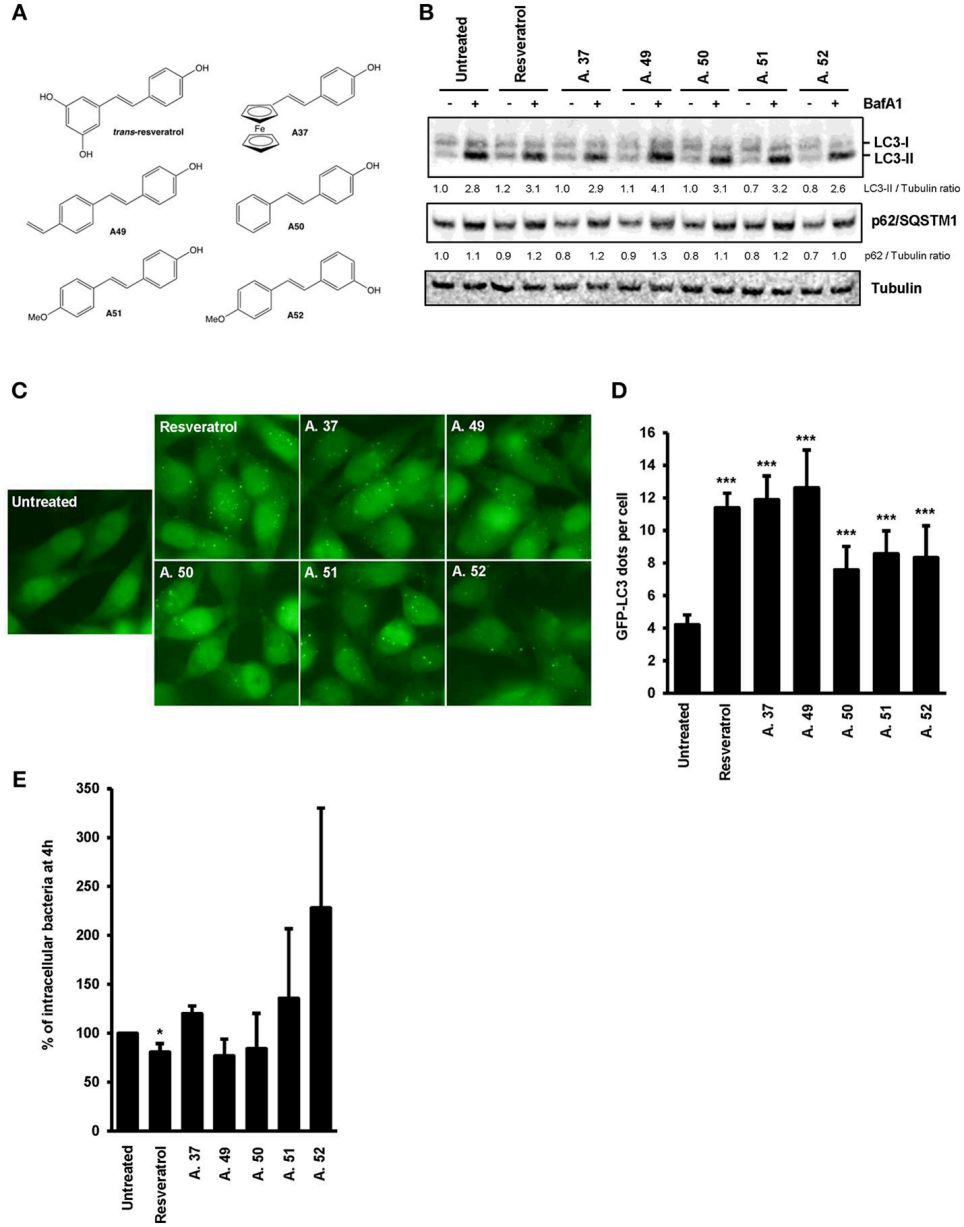
Comparative effects of resveratrol derivatives on the stimulation of autophagy response during infection. **(A)** Molecular structure of the *trans*-resveratrol and its derivatives (A.37, A.49, A.50, A.51, and A.52). **(B)** GFP-LC3 HeLa cells were untreated or treated for 20 h with 10 μM of *trans*-resveratrol or one of its derivatives. Bafilomycin A1 was added 2 h before protein extraction. Immunoblot analyses were performed using anti-LC3B, anti-p62/SQSTM1 and anti-Tubulin antibodies. Quantification of p62, LC3-II, and Tubulin was done and the ratios of p62/ Tubulin and LC3-II/Tubulin ratio were normalized to that obtained for untreated cells without BafA1, defined as 1.0 **(C)** Representative images of GFP-LC3-HeLa cells untreated or treated for 20 h with resveratrol or its derivatives. **(D)** Quantification of the number of GFP-LC3 positive dots per cell using the Icy software. Results are expressed as the percentage of GFP-LC3 positive dots per cell, relative to that obtained in untreated cells, taken as 100%. Each value is the mean of at least three independent experiments ± SEM. **(E)** GFP-LC3 HeLa cells were pre-treated or not with resveratrol or its derivatives at 10 μM for 20 h and then infected with *Salmonella*. Quantification was performed using the gentamicin protection assay. The number of intracellular bacteria was determined by CFU quantification at 30 min and 4 h post-infection. Results are expressed as the number of intracellular bacteria at 4 h post infection relative to that obtained at 30 min post infection, taken as 100%. Results obtained in untreated cells were defined as 100%. Data are means ± SEM of at least three independent experiments. *p < 0.05 and ***p < 0.001.

The *trans*-resveratrol molecule is hydrophobic and poorly soluble (39), thus we decided to synthetize derivatives (A.37, A.49, A.50, A.51, and A.52) that differ from the *trans*-resveratrol molecule by the nature and the position of substituents on phenyl rings (A49–A52) or by the replacement of a phenyl ring by a ferrocenyl ring (A37) (40). These modifications are predicting to improve the lipophilicity of these compounds in the cellular environment and thereby their biological effects inside the cell (41). These *trans*-resveratrol derivatives were tested for their efficiency to stimulate basal autophagy and to eliminate intracellular *Salmonella* in human epithelial cells, in comparison to the parent molecule (Figure 4). Under bafilomycin A1 treatment, higher LC3-II/Tubulin (LC3-II/T) ratios were observed in HeLa and HCT116 cells treated with the A49 derivative compared to cells treated with the parent molecule (Figure 4B, Figure S2), suggesting that this derivative is a more potent autophagy inducer than the *trans*-resveratrol molecule. This result was confirmed by analyzing the number of LC3 dots in cells by immunofluorescence (Figures 4C,D). In addition to the A.49 derivative, the A.37 derivative also induces an LC3-II accumulation in HCT116 cells compared to *trans*-resveratrol-treated cells (Figure S2). Cell treatment with the A.49 derivative tends to reduce the number of intracellular *Salmonella* at a similar level to that of *trans*-resveratrol-treated cells (Figure 4E). However, no effect on intracellular bacteria was observed in cells treated with the A.37 derivative (Figure 4E). This unexpected lack of effect on bacterial survival of the A.37 derivative might be due to the presence in this molecule of a ferrocenyl ring, that could have adverse effects on other cellular pathways (40). Interestingly, the resveratrol derivative A.52, displaying a decreased ability to accumulate the LC3-II protein in HeLa and HCT116 cells compared to the other resveratrol derivatives, increases the number of intracellular *Salmonella* compared to untreated or *trans*-resveratrol-treated cells. Altogether, these results indicate that modifications of the *trans*-resveratrol molecules, by changing the nature and the position of substituents on phenyl rings, can affect their impact on autophagy and clearance of invasive bacteria.

### *In vivo* Validation of Resveratrol-Induced Bacterial Clearance

**Figure 5:**
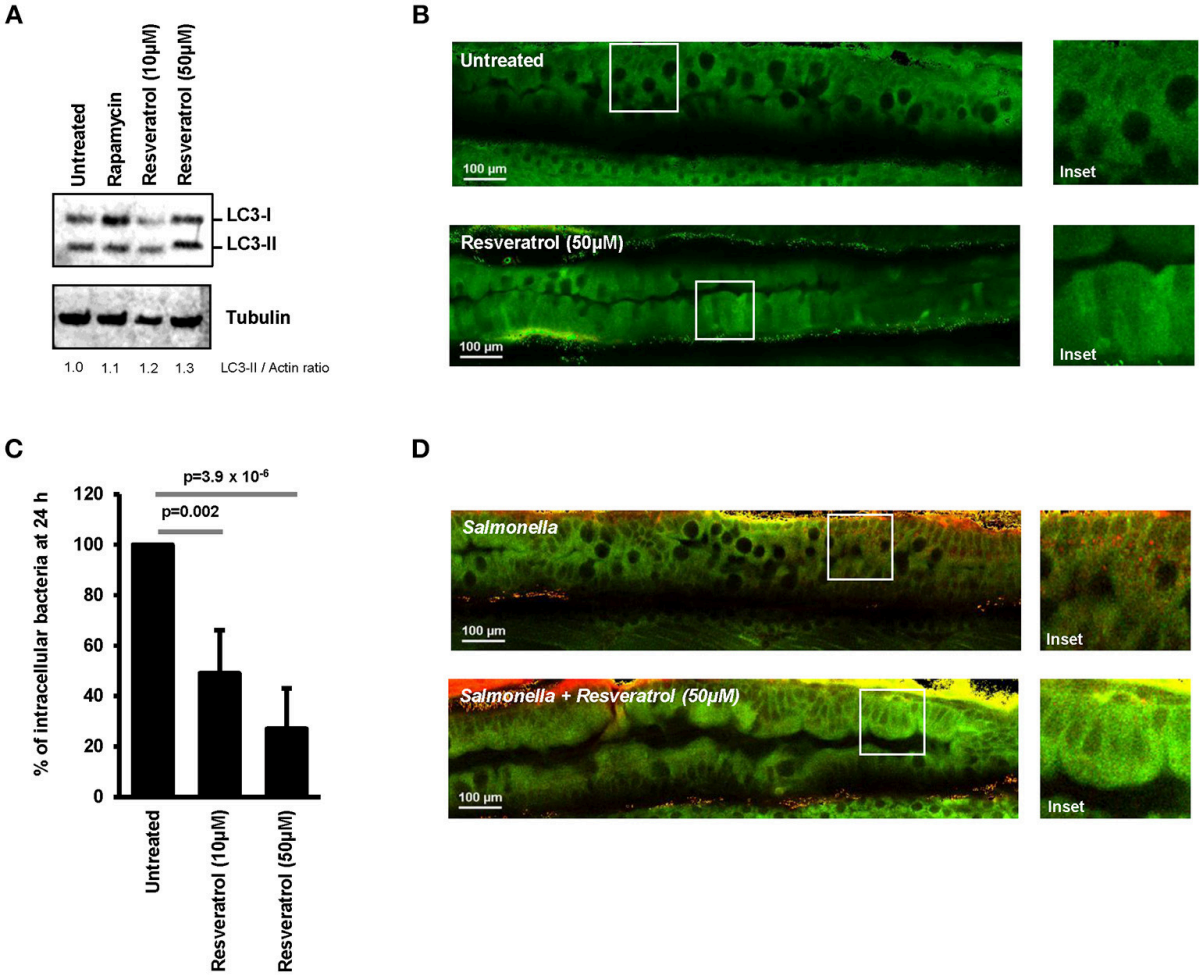
*Trans*-resveratrol induces autophagy and bacterial clearance in a zebrafish model. **(A)** Evaluation of LC3-II accumulation in zebrafish embryos untreated or treated with resveratrol (10 and 50 μM) and Rapamycin (40 μg/mL) by Western blot. Immunoblot analyses were performed using anti-LC3B and anti-Tubulin antibodies. Quantification of LC3-II and Tubulin was done and the ratios of LC3-II/Tubulin were normalized to that obtained for untreated embryos, defined as 1.0. **(B)** Representative images of the gut of GFP-LC3 zebrafish embryos untreated or treated for 24 h with 50 μM resveratrol. Images were acquired using a two-photon excitation confocal microscope. White square indicates inset area. **(C)** Counting of viable zebrafish-associated *Salmonella* at 24 h post-infection. Embryos were lysed, diluted and then plated onto TS agar plates. Data are means ± SEM of the six groups of 5 embryos of each condition. **(D)** Representative images of the gut of *Salmonella*-infected GFP-LC3 zebrafish embryos at 24 h post-infection. Zebrafish embryos were untreated or pre-treated for 24 h with 50 μM resveratrol before infection. Images were acquired using a two-photon excitation confocal microscope. White square indicates inset area.

Ability of *trans*-resveratrol to modulate autophagy activity and induce intracellular clearance of invasive bacteria was assessed *in vivo* by using transgenic GFP-LC3 expressing zebrafish embryos. This model is widely used to study the role of autophagy during bacterial infection (42–44). *Trans*-resveratrol was added to the water of GFP-LC3 zebrafish embryos (4 days post-fertilization) and autophagy was assessed using LC3 western blot and two-photon excitation microscopy to visualize the expression pattern of GFP-LC3 in the digestive tract of the embryos. Accordingly to literature, two doses resveratrol were tested, 10 and 50 μM (45, 46). A dose-dependent increase in the LC3-II protein level was observed in *trans*-resveratrol treated zebrafish embryos compared to controls demonstrating that *trans*-resveratrol stimulates autophagy *in vivo* (Figure 5A). In addition, *trans*-resveratrol was more potent than rapamycin for inducing autophagy in zebrafish embryos (Figure 5A). These results were confirmed by two-photon excitation microscopy observations. Whereas untreated zebrafish embryos harbored a weak and diffuse signal of the GFP-LC3 protein (green) in their digestive tract, a strong signal varying in intensity along the digestive tract was observed in embryos treated with *trans*-resveratrol (Figure 5B). Efficacy of *trans*-resveratrol to limit intracellular proliferation of invasive bacteria was also investigated in zebrafish embryos. Zebrafish embryos were infected for 24 h with *Salmonella* and the bacterial load in embryos was determined by CFU determination (Figure 5C) and microscopic observations (Figure 5D). As a consequence of *trans*-resveratrol treatment, the percentage of embryos-associated bacteria significantly decreased in a dose dependent manner (49 ± 17 and 27 ± 16% for embryos treated with 10 and 50 μM of *trans*-resveratrol, respectively) compared to untreated embryos (100%). A decrease in the number of embryos-associated *Salmonella* in the digestive tract of infected-embryos was also observed by immunostaining (Figure 5D). Altogether, these results showed that *trans*-resveratrol treatment is efficient to induce autophagy *in vivo* in zebrafish embryos and to limit invasion of the gastrointestinal tract by pathogens.

### Autophagy-Dependent Clearance of Intracellular Bacteria and Modulation of the Associated-Inflammatory Response in *Trans*-Resveratrol-Treated Macrophages

**Figure 6:**
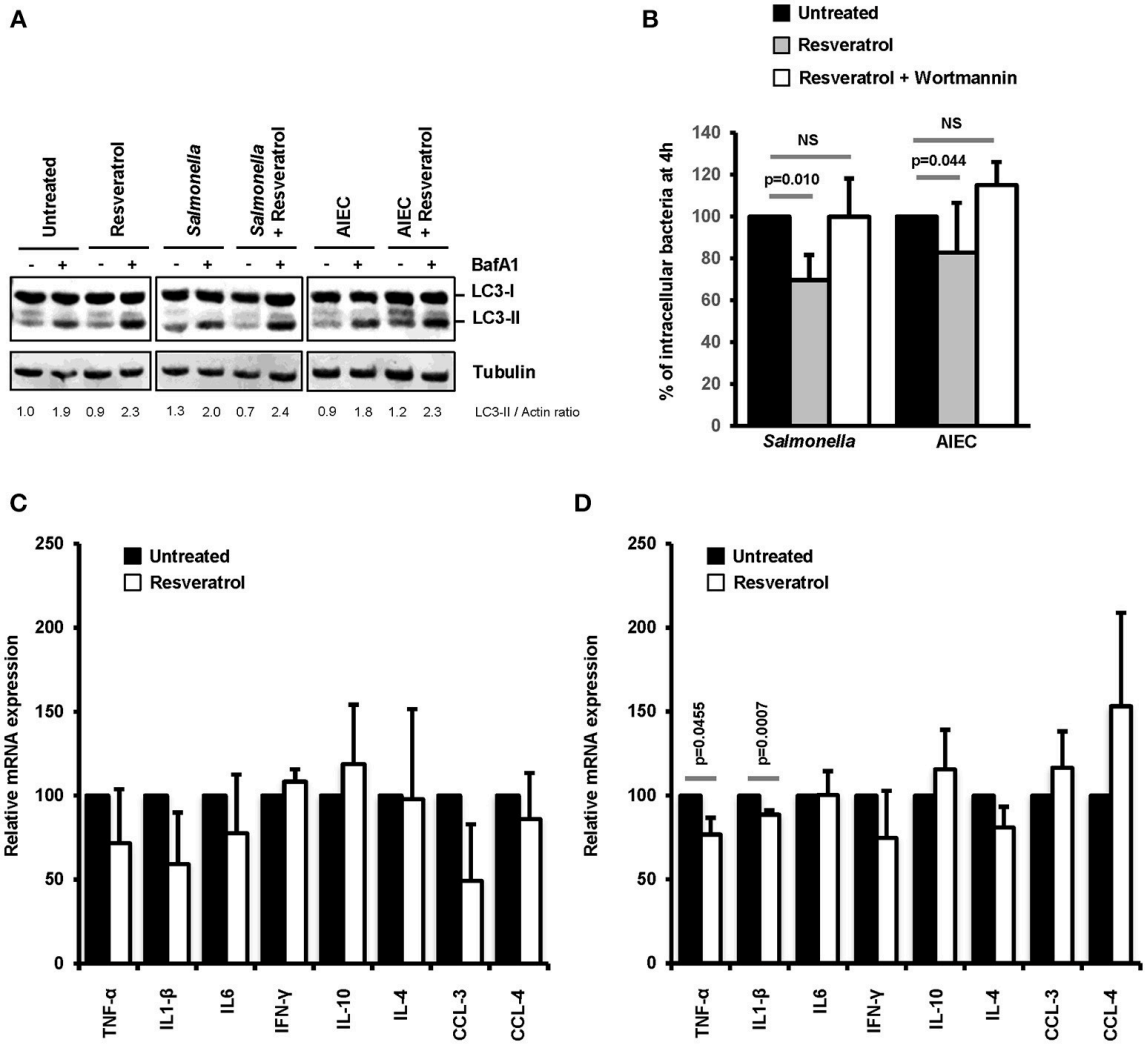
*Trans*-resveratrol treatment restrains the number of intramcrophagic bacteria and slows down the inflammatory response of infected cells. **(A)** RAW 264.7 macrophages were pre-treated or not for 20 h with *trans*-resveratrol at 10 μM and then infected with *Salmonella* or AIEC. Bafilomycin A1 (BafA1) at 100 nM was added to cells 30 min prior infection and maintained until protein extraction, at 1 h post-infection. Quantification of LC3-II and Actin was done and the ratios of LC3-II/Actin were normalized to that obtained for untreated cells without BafA1, defined as 1.0 **(B)** RAW 264.7 macrophages were pre-treated (gray bars) or not (black bars) for 20 h with resveratrol (10 μM), and then infected with *Salmonella* or AIEC. Autophagy was inhibited in cells with Wortmannin (white bars). Quantification was performed using the gentamicin protection assay. The number of intracellular bacteria was determined by CFU quantification at 30 min and 4 h post-infection. Results are expressed as the number of intracellular bacteria at 4 h post infection relative to that obtained at 30 min post infection, taken as 100%. Results obtained in untreated cells were defined as 100%. Data are means ± SEM of at least three independent experiments. **(C,D)** RAW 264.7 macrophages were pre-treated (gray bars) or not (black bars) for 20 h with resveratrol (10 μM), and then infected with *Salmonella* **(C)** or AIEC **(D)**. The mRNA levels of a set of pro-inflammatory cytokines (TNF-α, IL1-β, IL-6, IFN-γ, CCL-3, and CCL-4) and anti-inflammatory cytokines (IL-10 and IL-4) were quantified by RT-qPCR. The graphs show the average of three independent biological experiments using two replicates each. Each value is the mean of at least three independent experiments ± SEM.

The intestinal compartment includes a large number of immune cells that are involved in the maintenance of tolerance to commensal microbiota or food antigens and orchestrates immune defense against pathogens (47). Among these cells, macrophages represent the most abundant mononuclear phagocytes in the healthy gut lamina propria (48). On this basis, we decided to evaluate in macrophages the effects of *trans*-resveratrol treatment on autophagy and inflammation during bacterial infection. As shown in Figure 6A, *trans*-resveratrol treatment induces autophagy in murine RAW264.7 macrophages as evidenced by an increase in the LC3-II protein accumulation (LC3-II/Actin = 1.9), in comparison to untreated cells (LC3-II/Actin = 2.3). As observed in epithelial cells, *trans*-resveratrol treatment enhances the autophagic response induced by *Salmonella* and AIEC infection (Figure 6A). It also enables to significantly decrease the number of intracellular *Salmonella* and AIEC persisting within macrophages (Figure 6B). Autophagy induction and intracellular bacteria clearance by *trans*-resveratrol were confirmed in human THP-1 macrophages (Figures S3A–C). *Trans*-resveratrol-mediated clearance of intracellular bacteria relies on autophagy activation, since a concomitant treatment of macrophages with Wortmannin, an inhibitor of autophagy, totally abrogates this effect (Figure 6B).

Inflammatory responses, including those induced during infection, are regulated by autophagy (2). Cytokine mRNA expression in infected-macrophages treated or not with *trans*-resveratrol was measured. During both *Salmonella* and AIEC infection, *trans*-resveratrol pre-treatment tends to decrease the expression levels of several cytokine mRNA, notably those of the prototypical pro-inflammatory cytokines TNF-α and IL1-β in RAW264.7 and THP-1 macrophages (Figures 6C,D, Figures S3D,E). These results showed that *trans*-resveratrol treatment enables to concomitantly restrain intracellular replication of bacteria and slow down the inflammatory response.

## Discussion

Autophagy is central to maintain homeostasis and alteration of this cellular process is linked to numerous pathological conditions. Efforts have been made in the last decade to find pharmacological and nutritional autophagy modulators. Among dietary compounds, resveratrol, a polyphenolic stilbene, has been described as a potent inducer of basal autophagy and selective autophagy against mitochondria (mitophagy) (49, 50). In this study, we show that resveratrol is able to stimulate xenophagy, the selective form of autophagy that target invading bacteria and slow down the inflammatory response of infected cells. Autophagy-inducers are often well-characterized for their ability to stimulate a global autophagy response. However, less attention has been paid to their effects on selective forms of autophagy. Selective autophagy responses are named according to the targeted cargo such as mitophagy for mitochondria, pexophagy for peroxisomes, xenophagy for invading microbes, ribophagy for ribosomes, lipophagy for lipid droplets, and aggrephagy for aggregated proteins (51). Resveratrol has been originally described to induce a global autophagy response in ovarian cancer cells (50). This effect was then extensively confirmed in a wide variety of *in vitro* and *in vivo* models. Ability of resveratrol to stimulate selective forms of autophagy has been recently illustrated with mitophagy stimulation in resveratrol-treated cells in the context of Alzheimer's disease and during inflammatory responses (52, 53). However, to our knowledge, this is the first report describing induction of xenophagy by resveratrol. It has already been shown that resveratrol is able to restrict the number of intracellular *Chlamydia trachomatis* but the mechanism of action involved has not been identified (54). Besides, autophagy can restrict *C. trachomatis* survival suggesting that resveratrol might act on this cellular pathway to limit bacteria persistence in cells (55). In our study, we demonstrate that resveratrol enhances the autophagy-dependent intracellular clearance of two invasive bacteria well-described to be targeted by xenophagy, *S. Typhimurium*, and AIEC (18, 36). We assume that resveratrol could not only boost xenophagy against other pathogens sensitive to autophagy such as group A *Streptococci* or *Mycobacterium tuberculosis* (56, 57), but also overcome bacteria escape from autophagy as described for *Listeria monocytogenes, Shigella flexneri*, or *Legionella pneumophila* (58–60).

Chronic activation of autophagy using nutritional interventions may prevent or ameliorate the outcome of a wide range of human diseases associated with autophagy defects. Furthermore, nutritional approaches have the advantage of generally being less costly and with fewer side effects over long-term than pharmacological approaches. Caloric restriction (CR) can be considered as the most robust and reproducible way to sustain autophagy activity. Despite the tremendous potential of benefits to human health, some doubt may be cast on the capacity of people to be willing or to be able to follow a long CR, as suggested by studies exploring adherence of obese people to low-caloric diet (61). In addition, CR, if not properly done, can introduce a risk of nutrient deficiencies. One alternative to CR is to generate inter-meal fasting by allowing only two feeding periods per day, early and late in the diurnal cycle (62). The authors observed that, without reducing calories or altering the type of food consumed, mice that are exposed to inter-meal fasting harbor sustained autophagy in various organs and they are protected against age-associated metabolic defects. An alternative to CR is the development of dietary supplements that mimic biochemical and functional effects of CR (63). Interestingly, both resveratrol and CR activates sirtuin proteins and promote autophagy-dependent lifespan extension (33, 64). Our study demonstrates the ability of resveratrol to enhance autophagy-dependent intracellular bacteria clearance and slow down the inflammatory response of infected cells. Of note, resveratrol exhibits a more pronounced effect on bacteria clearance *in vivo*, compared to *in vitro* experiments, suggesting that, beyond autophagy, it might act on other cellular pathways involved in the control of infection. *In vitro*, starvation is also able to restrain the number of intracellular bacteria (18, 56). However, there are discrepancies regarding the impact of CR on infection outcome *in vivo*. In some cases, CR can exert a protective effect against bacterial infection (65), whereas it can weaken immune responses, especially in aged models (66, 67).

Low bioavailability of resveratrol at systemic level after oral administration is considered as a limiting factor in translating its effects from *in vitro*/*in vivo* experiments to humans (68, 69). In this study, we used resveratrol doses ranging from 10 to 50 μM to induce autophagy in culture cell lines or in zebrafish embryos. One practical question will be now to determine whether this range of concentration can be reached in tissues to enable autophagy stimulation. A wide range of resveratrol doses, from 30 nM to 100 μM, are found in the literature in studies that aim at testing its effects on various cellular processes (29). Most of the data on pharmacokinetics and bioactivity of resveratrol has been generated in rodent models. Rats receiving resveratrol doses from 2 to 50 mg/kg or mice receiving doses from 2 to 240 mg/kg *per os* reached a micromolar range concentration in serum (29), which is entirely compatible with resveratrol doses used *in vitro* to stimulate autophagy. In human, a phase I pharmacokinetic study has demonstrated that a single high dose of resveratrol (5 g), that did not cause side effects, allows to reach a 2.4 μM concentration at plasma level in healthy volunteers (68). Interestingly, a study has shown that after resveratrol administration to patients with colorectal cancer, resveratrol metabolites levels (sulfate and glucuronides metabolites) were very high in colorectal tissues (70). In fact, the authors shown that resveratrol is delivered to target tissues in a stable sulfate-conjugated form and that the parent compound is gradually regenerated in selected cells and may give rise to the beneficial effects *in vivo* (71, 72). This importance of sulfate-conjugated metabolites result from an extensive metabolization of resveratrol in the liver, an organ where resveratrol is glucuronidated and sulfated by the P450 isoenzyme CYP1B1 and sulfotransferase (73). Conversely, sulfatases can regenerate the aglycone molecule from sulfated resveratrol inside the cells (72). Importance of this regeneration phenomenon in the intracellular bioavailability remains to be determined. In order to favor resveratrol bioactivity and stability, resveratrol derivatives can be developed. In this study, we tested whether resveratrol derivatives, mostly modified in the nature and the position of substituents on phenyl rings, still conserved their ability to stimulate autophagy. All derivatives remain able to stimulate autophagy at a level comparable to those observed for the parent molecule, suggesting that resveratrol molecule can be modified to improve its bioavailability without affecting its autophagy inducer property. Further studies will be necessary, using a wider range of derivatives, to select a derivative combining a high bioavailability and a potent stimulatory effect on autophagy.

In conclusion, the data presented here describe a novel role for resveratrol in stimulating xenophagy, a selective form of autophagy dedicated to intracellular bacteria clearance. In resveratrol-treated cells, an increased number of the invasive bacteria *Salmonella* and Crohn's disease-associated AIEC are targeted to autophagy demonstrating that this micronutrient boost innate immunity. Control of intracellular bacteria by xenophagy is associated with a slowdown of the inflammatory response. These findings offer the intriguing possibility of using autophagy-inducing nutrients to stimulate and/or restore this process to prevent infectious and immune disorders associated with autophagy defects.

## Materials and Methods

### Bacterial Strains and Culture Conditions

*Salmonella enterica* serovar Typhimurium reference strain C5 and the Adherent-Invasive *Escherichia coli* (AIEC) reference strain LF82, which has been isolated from a chronic ileal lesion of a CD patient (74), were grown overnight in Tryptic Soy broth (TSB) at 37°C from a frozen stock (under agitation for *S. Typhimurium*).

### Cellular Models and Culture Conditions

The human intestinal epithelial cells HCT116, the human cervical epithelial cells HeLa, and Mouse Embryonic Fibroblasts (MEFs) Wild-Type (WT), knock-out for the *Atg5* (Atg5 KO), or *Atg7* (Atg7 KO) genes were cultured in Dulbecco's Modified Eagle Medium (DMEM) with L-glutamine (Gibco) supplemented with 10% fetal bovine serum (Eurobio) and 1% antibiotics (Penicillin/streptomycin; Eurobio). Murine macrophages RAW 264.7 and human monocytic THP-1 cells were cultured in RPMI medium 1640 (Gibco) supplemented with 10% fetal bovine serum and 1% antibiotics (Penicillin/streptomycin). Cells were maintained in an atmosphere containing 5% CO_2_ at 37°C. When required (GFP-LC3-HeLa and mRFP-GFP-LC3-HeLa), 500 μg/ml of G418 (Sigma) was added in the medium. For experiments, cells were seeded 48 h before in medium without antibiotics at 2 × 105 cells per well in 24-wells tissue culture plates. THP-1 monocytes were differentiated in macrophages by treating them for 18 h with 20 ng/ml of phorbol myristate acetate (PMA, Sigma) before infection. All cell lines have been routinely tested for mycoplasma contamination using the PCR Mycoplasma Test Kit II (PromoKine).

### Antibodies and Reagents

For immunoblotting (IB) and immunofluorescence (IF) experiments the following antibodies were used: rabbit polyclonal anti-LC3B (7543, Sigma, IB dilution 1:1000, IF dilution: 1:200), rabbit polyclonal anti-Actin (A2066, Sigma, IB dilution 1:2000) and mouse monoclonal anti-Tubulin (T5168, Sigma, IB dilution 1:5000), mouse monoclonal anti-p62/SQSTM1 (#610833, Sigma, IB dilution 1:2000), mouse monoclonal antibody anti-LPS from *Salmonella* (5D12A, Bio-rad, IF dilution: 1:500). *Trans*-resveratrol, DAPI and gentamicin were purchased from Sigma. Bafilomycin A1, Wortmannin and Rapamycin were purchased from LC laboratories.

### Invasion Assay

For *S. Typhimurium*, overnight cultures were subcultured (1:30) in fresh TSB and incubated for 3 h at 37°C before infection. For AIEC bacteria, an overnight culture was used for infection. Bacterial concentrations were estimated by measuring the optical density at 600 nm (1 OD_600_ = 5 × 10^8^ cells per ml). Bacteria were harvested at 2000 g for 10 min and re-suspended in a proper volume of PBS to allow infection of host cells at a MOI (multiplicity of infection) of X. Invasion assays were performed using the gentamicin protection assay as described previously (18). Briefly, cell monolayers were infected with bacteria at MOI (multiplicity of infection) of 100. For assays in fibroblasts or epithelial cells, infected cells were centrifuged 10 min at 1,000 g to favor bacteria/cells contact. Thereafter, infected cells were incubated 10 min (*S. Typhimurium*) or 1 h (AIEC) at 37°C in an atmosphere containing 5% CO_2_, then cells were washed twice with PBS, and fresh cell culture medium containing 50 mg/mL of gentamicin was added for 30 min (determination of cell invasion by bacteria) or 4 h (determination of the number of intracellular bacteria that survive within cells). The same method was used in macrophages except that the incubation period for AIEC was 10 min. To determine the number of intracellular bacteria, cell monolayers were washed twice with PBS and lysed with 1% Triton X-100 (Sigma) in PBS. Samples were then mixed, diluted and plated onto TC agar plates to determine the number of colony forming unit (CFU) recovered from the lysed monolayers.

### Autophagy Modulation

Autophagy was blocked by treating cells with culture medium containing Wortmannin, an inhibitor of phosphatidylinositol 3-kinase (PI3K), at 100 nM for 30 min prior the infection. For autophagy flux assays, cells were pre-treated with Bafilomycin A1, an inhibitor of vacuolar-type H^+^ ATPases, at 100 nM for 30 min prior the infection and Bafilomycin A1 was then maintained in the cell culture medium all along the experiment. Autophagy induction in GFP-LC3 zebrafish embryos was performed by adding rapamycin, a mammalian target of rapamycin (mTOR) inhibitor, at 40 μg/ml in the fish water during 24 h.

### Immunoblot Analysis

Cells were lysed in Laemmli buffer (2% SDS, 5% 2-mercapto-ethanol, 10% glycerol, 0.002% bromophenol blue, 0.125 M Tris-HCl). Cell lysates were sonicated for 5 min and proteins were denatured by heating at 95°C for 5 min. For protein extraction from zebrafish embryos, fifteen embryos per condition were washed and pooled in 50 μL of lysis buffer (50 mM Tris-HCl pH8.0, 0.1 mM EDTA, 200 mM NaCl, 0.5% NP40, 10% glycerol) containing 1X protease inhibitors (Roche). Each pool of embryos was lysed by using ceramic beads (Lysing Matrix D, MP Biomedicals) and the Precellys 24 lysing system (Bertin Technologies). Proteins were loaded on 4–15% mini-Protean TGX gradient protein gels (Bio-Rad), transferred on nitrocellulose membrane (Mix molecular weight program, Trans-blot turbo, Bio-rad). Membranes were saturated for 1 h (Odyssey Blocking Buffer TBS, Li-Cor) and then immunoblotted overnight at 4°C with the indicated primary antibodies diluted in PBS. Anti-rabbit and anti-mouse antibodies conjugated with IR800 or IR680 dyes were used as secondary antibodies, diluted in PBS and incubated with the membranes for 45 min at room temperature. The infrared signal was integrated using an infrared imaging system (LI-COR Odyssey). The bands intensities were determined using the software associated with the Odyssey system (Image studio).

### Fluorescence Microscopy

Cells were fixed for 10 min at room temperature with 4% paraformaldehyde (PFA) in PBS and permeabilized and saturated for 20 min at room temperature in blocking buffer (10% FBS, 0.1% saponin PBS). Next, cells were incubated at room temperature for 2 h with the indicated primary antibodies diluted in blocking buffer, washed three times in PBS and then incubated for 1 h at room temperature with alexa fluor conjugated secondary antibodies (Thermo Scientific) and DAPI. Images were acquired using fluorescent microscope (Axiovision Zeiss). The number of LC3 dots per cell were counted in at least 100 cells using the spot detector plugin of Icy software (75). Each microscopy image is representative of at least three independent experiments.

For *in vivo* imaging, 5 GFP-LC3 zebrafish embryos were pooled by conditions, washed in sterile water and fixed in 200 μL of formalin for 24 h at 4°C. Embryos were permeabilized and saturated for 30 min in blocking buffer. *S. Typhimurium* was labeled using anti-LPS antibody. Zebrafish embryos were imaging on a two-photon excitation microscope (Nikon A1-MP, DImaCell platform).

### RNA Isolation and qPCR Analysis

Total RNAs were extracted using TRIzol reagent (Sigma) following manufacturer's instructions and quantified using a NanoDrop spectrophotometer (Thermo Scientific). cDNAs were generated from 1 μg total RNAs using High-Capacity cDNA Reverse Transcription Kit (Applied Biosystems). The mRNA levels were determined by quantitative real-time PCR analysis using iTaq universal SYBR Green supermix (Bio-rad) and the primer sets listed in Table 1. ΔCt values were calculated using the Ct values from the amplification of endogenous glyceraldehyde-3-phosphate dehydrogenase (*Gapdh*) and hypoxanthine-guanine phosphoribosyltransferase (*Hprt*) mRNAs. Quantitative real-time PCR was performed on the CFX96 PCR system (Bio-rad).

**Table 1:** List of primer sequences used for RT-PCR analysis in this study.

**Species**	**Target mRNA**	**Forward primer**	**Reverse primer**
Mus musculus	*Tnf-α*	GGTGCCTATGTCTCAGCCTC	GCTCCTCCACTTGGTGGTTT
	*Il1-β*	GCCACCTTTTGACAGTGATGAG	GACAGCCCAGGTCAAAGGTT
	*Il-6*	TGATGGATGCTACCAAACTGGA	TGTGACTCCAGCTTATCTCTTGG
	*Ifn-γ*	TCATTGAATGCTTGGCGCTG	AGCAAGGCGAAAAAGGATGC
	*Il-10*	TAACTGCACCCACTTCCCAG	AAGGCTTGGCAACCCAAGTA
	*Il-4*	CTTGGAAGCCCTACAGACGA	GATGGATGTGCCAAACGTCC
	*Ccl-3*	CGTGGAATCTTCCGGCTGTA	TACAAGCAGCAGCGAGTACC
	*Ccl-4*	TTCTGTGCTCCAGGGTTCTC	CTCACTGGGGTTAGCACAGA
	*Hprt*	CAGTCCCAGCGTCGTGATTA	TGGCCTCCCATCTCCTTCAT
	*Gapdh*	ACCCAGAAGACTGTGGATGG	ACACATTGGGGGTAGGAACA
Homo sapiens	*Tnf-α*	ACTTTGGAGTGATCGGCCC	CATTGGCCAGGAGGGCATT
	*Il1-β*	GCCAATCTTCATTGCTCAAGTGT	GGTCGGAGATTCGTAGCTGG
	*Il-8*	TCCTGATTTCTGCAGCTCTGT	CCAGACAGAGCTCTCTTCCA
	*Ccl-3*	TGGCTCTCTGCAACCAGTTCT	CCGGCTTCGCTTGGTTAGG
	*Ccl-5*	CGTGCCCACATCAAGGAGTA	CCAGACTTGCTGTCCCTCTC
	*Il-10*	CGAGATGCCTTCAGCAGAGT	CGCCTTGATGTCTGGGTCTT
	*Ulk1*	GACGACTTCGTCATGGTCCC	CACTGCACATCAGGCTGTCT
	*Wipi1*	GGACTGCACATGAAATCCCG	CTAGGCAAACCAGCAGCCT
	*Atg5*	TCCCTCTTGGGGTACATGTCT	CGTCCAAACCACACATCTCG
	*Atg16L1*	AGGAGATCATCCTGCAATAACAA	TCCATGTGCCATCATGTCCG
	*p62/SQSTM1*	CATTGCGGAGCCTCATCTCC	TCCTCGTCACTGGAAAAGGC
	*Ndp52*	TCACCCAGCATTTCATCCCT	CCTTGGCTCCTCCATTTGAGT
	*Nbr1*	ACCATCATGGGAGCAGCATT	AAGGAATGACAGCAAGCCCC
	*Optineurin*	CCTGGGCCCACGAGAAC	TCTTTGGCCTCCTTGAGTGC
	*Hprt*	TTGCTTTCCTTGGTCAGGCA	ATCCAACACTTCGTGGGGTC
	*Gapdh*	AAGCCTGCCGGTGACTAAC	GTTAAAAGCAGCCCTGGTGAC

### Zebrafish Maintenance

Zebrafish (*Danio rerio*) were maintained in a recirculating aquaculture system (Müller and Pfleger, Germany). Photoperiod was 14 L:10 D (light:dark) and the mean ranges for conductivity, pH, and temperature in the system were 600–700 μS, pH 8.0 and 26–28°C, respectively. Experiments were performed using the transgenic GFP-Lc3 line [a generous gift of Daniel Klionsy (76)].

### Infection of Zebrafish Embryos

Natural breeding eggs were collected immediately after hatching and transferred to a sterile dish with sterilized egg water containing antibiotics (ampicillin and kanamycin, Sigma). Unfertilized embryos were removed timely over the next few days. At 4 dpf, embryos were treated with *trans*-resveratrol for 24 h with 10 or 50 μM doses. In parallel, overnight *S. Typhimurium* culture was washed with sterile egg water and adjusted to OD600 nm equal to 0.6 (10^9^ CFU/mL). Infection was performed by oral immersion for 24 h at 28°C. Thirty embryos were collected for each group (untreated or treated with 10 and 50 μM *trans*-resveratrol). Groups were separated into six sub-groups and each five embryos were anesthetized with tricaïne (Sigma) at 24 h post-infection. Each pool of 5 embryos were treated with 100 μl of PBS-Triton 1% for 15 min. Embryos were then placed in tubes containing ceramic beads and lysed using the Precellys 24 lysing system (Bertin Technologies). Serial dilutions were performed and diluted samples were plated on TS agar plates Results are presented as mean log10 CFU ± SE per five embryos.

### Statistical Analysis

All data are expressed as means. Error bars indicate SEM or Sd. Student's *t*-test was used for comparison of the two groups of data. All experiments were performed at least three times. A *P*-value less than or equal to 0.05 was considered statistically significant.

## Ethics Statement

All experiments were executed in strict accordance with the European Council Directive (2010/63/EC). According to the EU Directive on the protection of animals used for scientific purposes (2010/63/EU) and the Commission Implementing Decision (2012/707/EU), fish are non-protected animals until the stage of free feeding; this limit was set at 120 h post fertilization (hpf) for zebrafish. No specific ethics approval was required for this project, as all zebrafish embryos used in this study were at maximum 120 hpf old.

## Author Contributions

JA and PL designed the experiments. JA performed most of the experiments. VA, DD, JC, M-AB, and FD provided technical assistance. JL and DV-F conceived and synthetized resveratrol derivatives. PW assisted with two-photon microscopy. AR, PL, and JG conceived and supervised the study. PL and JA wrote the manuscript.

### Conflict of Interest Statement

The authors declare that the research was conducted in the absence of any commercial or financial relationships that could be construed as a potential conflict of interest.

## Supplementary Material

Figure S1 **(A)** HeLa and **(B)** HCT116 cells were untreated (black bars) or pre-treated for 20 h with resveratrol at 10 μM (white bars) and mRNA levels of various autophagy-related genes were measured by RT-qPCR. The graphs show the mean of three independent biological experiments using two replicates each. Each value is the mean of at least three independent experiments ± SEM. ^*^*p* < 0.05, ^**^*p* < 0.01, and ^***^*p* < 0.001.

Figure S2 **(A)** HCT116 cells were treated or not for 20 h with 10 μM of *trans*-resveratrol or one of its derivatives (A.37, A.49, A.50, A.51, and A.52). Bafilomycin A1 (BafA1) was added 1 h before protein extraction. Immunoblot analyses were performed using anti-LC3B and anti-Tubulin antibodies. Quantification of LC3-II and Tubulin was done and the ratios of LC3-II/Tubulin were normalized to that obtained for untreated cells without BafA1, defined as 1.0. **(B)** HCT116 cells were treated or not for 20 h with 10 μM of *trans*-resveratrol or one of its derivatives. The number of intracellular bacteria was determined by CFU quantification at 30 min and 4 h post-infection. Results are expressed as the number of intracellular bacteria at 4 h post infection relative to that obtained at 30 min post infection, taken as 100%. Results obtained in untreated cells were defined as 100%. Data are means ± SEM of at least three independent experiments.

Figure S3 **(A)** Representative images of *Salmonella*- and AIEC-infected THP-1 macrophages at 1 h post-infection, with or without a 20 h resveratrol pre-treatment. Samples were processed for LC3B immunostaining (red) and nuclei staining (DAPI, blue). **(B)** Quantification of the number of LC3B positive vacuoles per macrophage using the Icy software. Results are expressed as the percentage of LC3B positive dots per cell, relative to that obtained in untreated cells, taken as 100%. Each value is the mean of at least three independent experiments ± SEM. **(C)** THP-1 macrophages were treated (white bars) or not (black bars) for 20 h with *trans*-resveratrol at 10 μM, and then infected with *Salmonella* or AIEC. The number of intracellular bacteria was determined by CFU quantification at 30 min and 4 h post-infection. Results are expressed as the number of intracellular bacteria at 4h post infection relative to that obtained at 30 min post infection, taken as 100%. Results obtained in untreated cells were defined as 100%. Data are means ± SEM of at least three independent experiments. **(D,E)** THP-1 macrophages were treated (white bars) or not (black bars) for 20 h with *trans*-resveratrol at 10 μM, and then infected with *Salmonella* **(D)** or AIEC **(E)**. The mRNA levels of pro-inflammatory cytokines (TNF-α, IL1-β, IL-8, CCL-3, and CCL-5) and the anti-inflammatory cytokines IL-10 were measured by RT-qPCR. The graphs show the average of three independent biological experiments using two replicates each. Each value is the mean of at least three independent experiments ± SEM. **(E)** Same as in **(D)** but macrophages were infected with AIEC.
