# Supplementary figures and images for: Resveratrol-Induced Xenophagy Promotes Intracellular Bacteria Clearance in Intestinal Epithelial Cells and Macrophages
Source: Front Immunol. 2019 Jan 14;9:3149. doi: 10.3389/fimmu.2018.03149 (PMC6339935; doi:10.3389/fimmu.2018.03149)

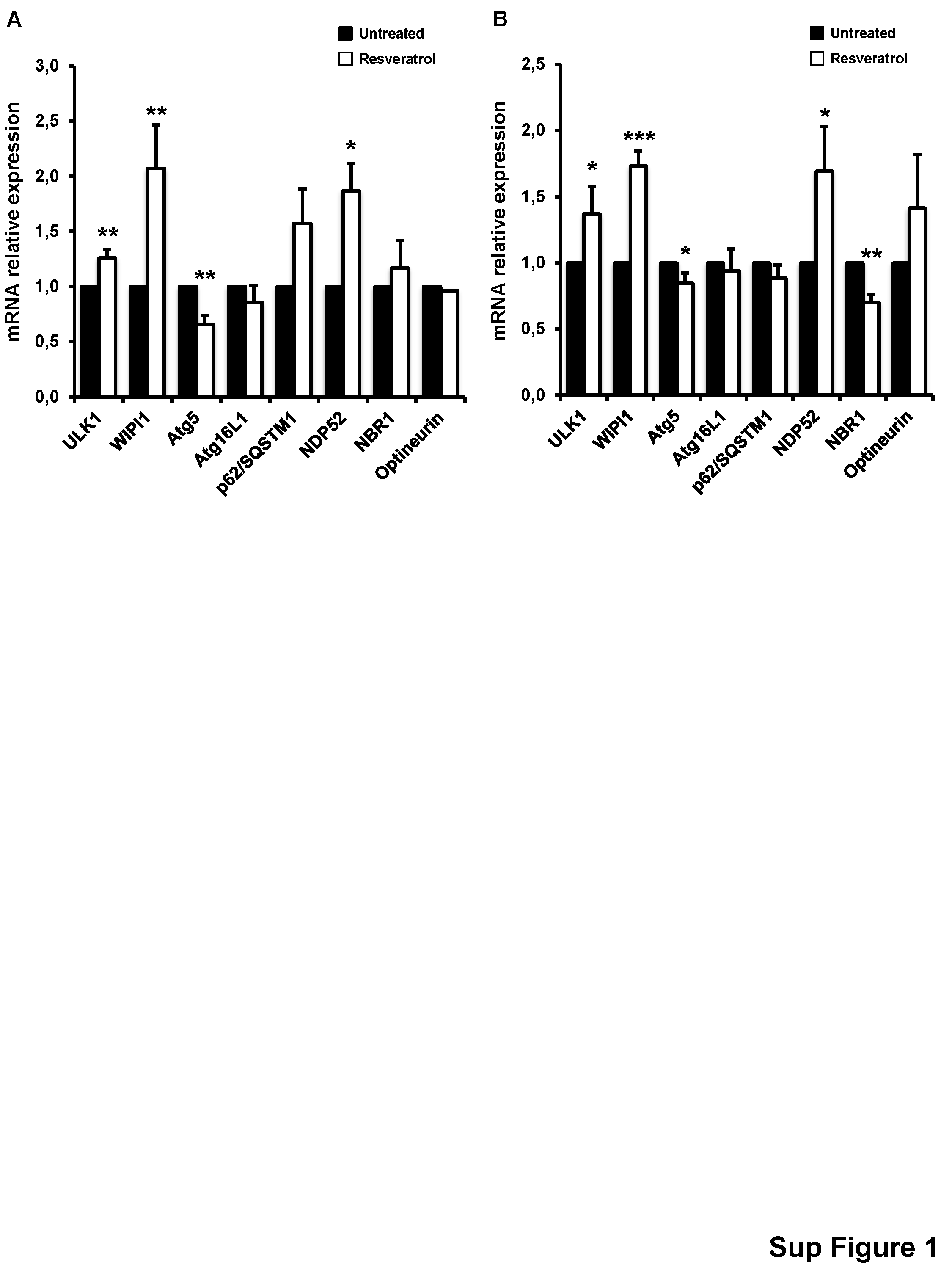

Supplement: Figure S1 — (A) HeLa and (B) HCT116 cells were untreated (black bars) or pre-treated for 20 h with resveratrol at 10 μM (white bars) and mRNA levels of various autophagy-related genes were measured by RT-qPCR. The graphs show the mean of three independent biological experiments using two replicates each. Each value is the mean of at least three independent experiments ± SEM. *p < 0.05, **p < 0.01, and ***p < 0.001. [file Image_1.TIF]

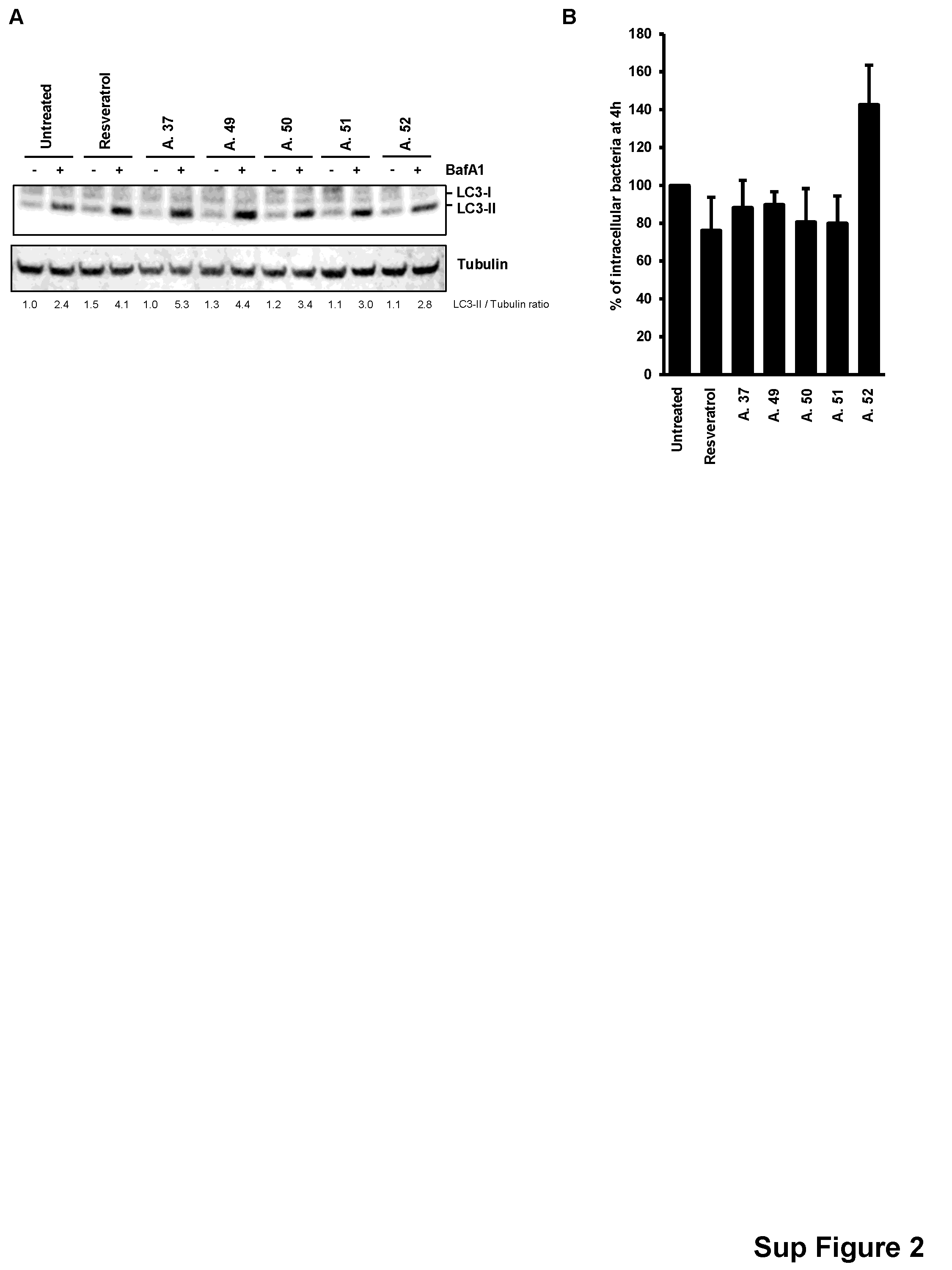

Supplement: Figure S2 — (A) HCT116 cells were treated or not for 20 h with 10 μM of trans-resveratrol or one of its derivatives (A.37, A.49, A.50, A.51, and A.52). Bafilomycin A1 (BafA1) was added 1 h before protein extraction. Immunoblot analyses were performed using anti-LC3B and anti-Tubulin antibodies. Quantification of LC3-II and Tubulin was done and the ratios of LC3-II/Tubulin were normalized to that obtained for untreated cells without BafA1, defined as 1.0. (B) HCT116 cells were treated or not for 20 h with 10 μM of trans-resveratrol or one of its derivatives. The number of intracellular bacteria was determined by CFU quantification at 30 min and 4 h post-infection. Results are expressed as the number of intracellular bacteria at 4 h post infection relative to that obtained at 30 min post infection, taken as 100%. Results obtained in untreated cells were defined as 100%. Data are means ± SEM of at least three independent experiments. [file Image_2.TIF]

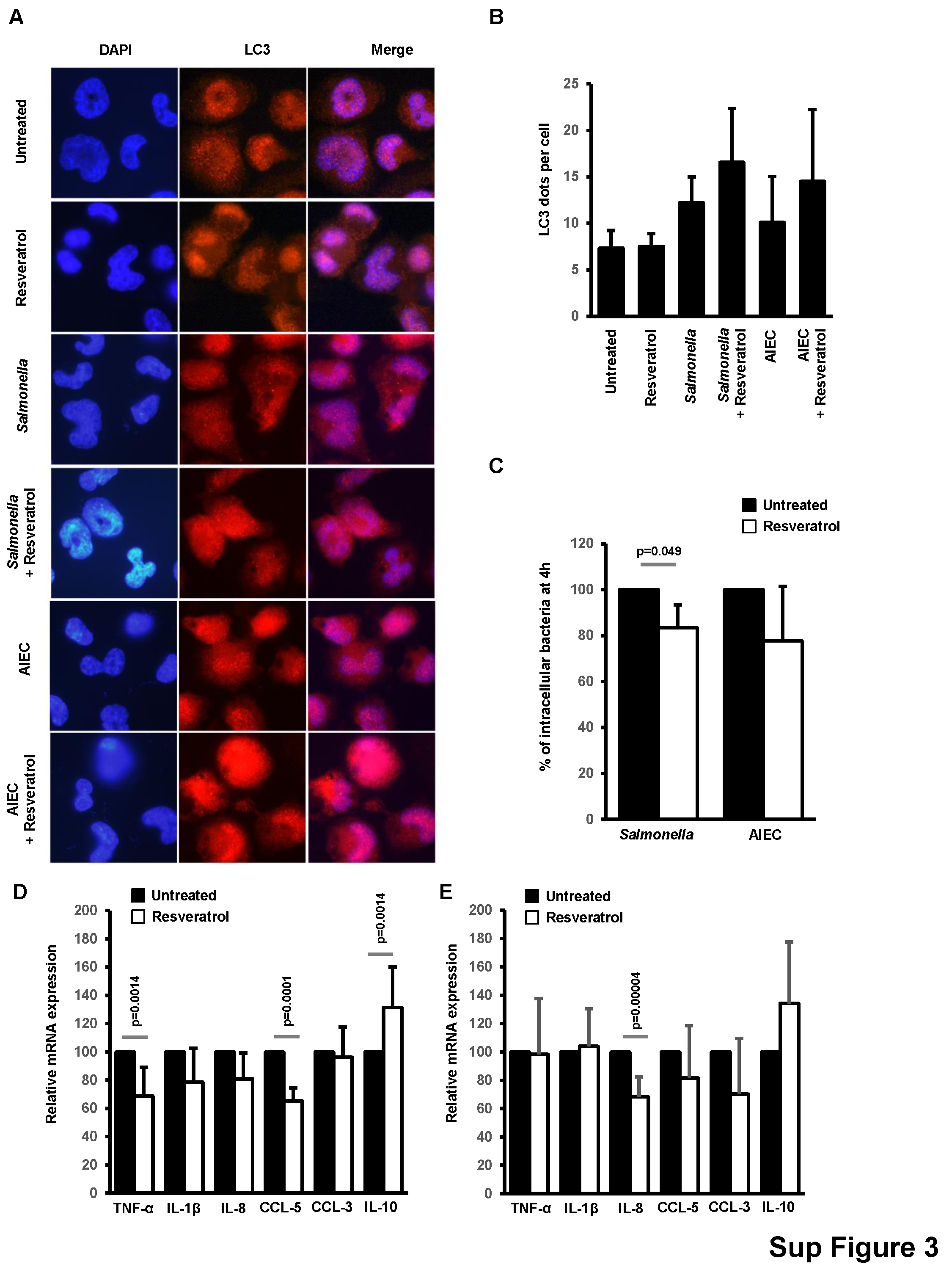

Supplement: Figure S3 — (A) Representative images of Salmonella- and AIEC-infected THP-1 macrophages at 1 h post-infection, with or without a 20 h resveratrol pre-treatment. Samples were processed for LC3B immunostaining (red) and nuclei staining (DAPI, blue). (B) Quantification of the number of LC3B positive vacuoles per macrophage using the Icy software. Results are expressed as the percentage of LC3B positive dots per cell, relative to that obtained in untreated cells, taken as 100%. Each value is the mean of at least three independent experiments ± SEM. (C) THP-1 macrophages were treated (white bars) or not (black bars) for 20 h with trans-resveratrol at 10 μM, and then infected with Salmonella or AIEC. The number of intracellular bacteria was determined by CFU quantification at 30 min and 4 h post-infection. Results are expressed as the number of intracellular bacteria at 4h post infection relative to that obtained at 30 min post infection, taken as 100%. Results obtained in untreated cells were defined as 100%. Data are means ± SEM of at least three independent experiments. (D,E) THP-1 macrophages were treated (white bars) or not (black bars) for 20 h with trans-resveratrol at 10 μM, and then infected with Salmonella (D) or AIEC (E). The mRNA levels of pro-inflammatory cytokines (TNF-α, IL1-β, IL-8, CCL-3, and CCL-5) and the anti-inflammatory cytokines IL-10 were measured by RT-qPCR. The graphs show the average of three independent biological experiments using two replicates each. Each value is the mean of at least three independent experiments ± SEM. (E) Same as in (D) but macrophages were infected with AIEC. [file Image_3.TIF]
